# Plug-and-Push Embolisation of a Tentorial Dural Arteriovenous Fistula Using Squid-18 and Squid-12

**DOI:** 10.7759/cureus.106227

**Published:** 2026-03-31

**Authors:** Josiah Cho, Arvindh Sekaran, Nathan Chan

**Affiliations:** 1 Medicine, Addenbrooke's Hospital, Cambridge University Hospitals NHS Foundation Trust, Cambridge, GBR; 2 Interventional Neuroradiology, Addenbrooke's Hospital, Cambridge University Hospitals NHS Foundation Trust, Cambridge, GBR

**Keywords:** dural arteriovenous fistula, endovascular embolisation, interventional neuroradiology, liquid embolic agents, squid

## Abstract

Dural arteriovenous fistulas (DAVFs) are abnormal shunts between meningeal arteries and dural venous sinuses or cortical veins. Lesions with cortical venous reflux carry a substantial risk of haemorrhage or non-haemorrhagic neurological deficit and therefore require prompt treatment. Endovascular embolisation using ethylene-vinyl alcohol copolymer (EVOH)-based liquid embolic agents is widely employed. Squid, a newer agent available in different viscosities, includes Squid-18 and the lower-viscosity Squid-12, which may improve distal penetration during embolisation. However, reports describing staged viscosity-based embolisation using Squid with the plug-and-push technique remain limited.

A male in his 70s presented with several months of progressive unsteadiness culminating in a fall. Computed tomography demonstrated cerebellar calcifications without acute intracranial pathology, while MRI showed dilated cerebellar and perimesencephalic veins. Digital subtraction angiography confirmed a Cognard grade IV tentorial DAVF near the superior vermis, supplied by branches of both external carotid arteries, the left internal carotid artery, and the right vertebral artery, with cortical venous drainage into ectatic cerebellar veins. Endovascular embolisation was performed via the distal temporal branch of the left middle meningeal artery using a Marathon microcatheter. A plug-and-push technique was employed, with formation of an anti-reflux plug using Squid-18, followed by injection of Squid-12 to facilitate deeper penetration of the fistulous network.

Complete angiographic occlusion was achieved without complications after a 90-minute embolisation. The patient’s mobility improved significantly, and eight-month follow-up angiography demonstrated durable resolution. This case highlights the feasibility of staged viscosity-based embolisation using Squid-18 and Squid-12 to achieve effective occlusion of a complex tentorial DAVF.

## Introduction

Dural arteriovenous fistulas (DAVFs), characterised by abnormal communications between meningeal arterial branches and the dural venous sinuses or cortical veins, demonstrate a potentially aggressive clinical course that is strongly influenced by the pattern of venous drainage. Under normal circumstances within the dura, blood flows from arteries into small capillaries and then into veins at low pressure. In DAVFs, this capillary network is bypassed, causing high-pressure arterial blood to flow directly into veins. This can lead to increased venous pressure, vessel dilation, and impaired drainage of blood from the brain. Those DAVFs exhibiting cortical venous reflux carry an annual risk of haemorrhage or non-haemorrhagic neurological deficit of approximately 8-15% [1,2]. Clinical intervention, most commonly via an endovascular approach, is therefore critical to prevent these sequelae in high-grade DAVFs. Several liquid embolic agents are available for endovascular intervention, including ethylene-vinyl alcohol copolymer (EVOH)-based agents such as Onyx and Squid, as well as Precipitating Hydrophobic Injectable Liquid (PHIL), a hydrophobic polymer bound to an iodine-containing radiopaque compound. Squid, a newer EVOH-based liquid embolic agent, offers a lower-viscosity option (Squid-12) compared to Onyx, which enhances its flow properties during embolisation, with viscosity being quantified in centipoise. Squid is designed with smaller tantalum particles, which allow for a more homogeneous suspension and provide more uniform radiopacity on fluoroscopy. Squid's smaller tantalum particles also lead to a slower sedimentation rate compared to standard-grain tantalum agents such as Onyx. This allows for longer visibility during extended injection times and reduces the risk of syringe or microcatheter occlusion [3]. Previously, the use of Onyx with plug formation and controlled injection has shown success in occluding DAVFs [4]. However, at present, the literature surrounding this approach using differing viscosities of Squid is limited. In this case report, we illustrate the application of Squid-18 and Squid-12 for treating a Cognard grade IV tentorial DAVF using this technique.

## Case presentation

**Figure 1:**
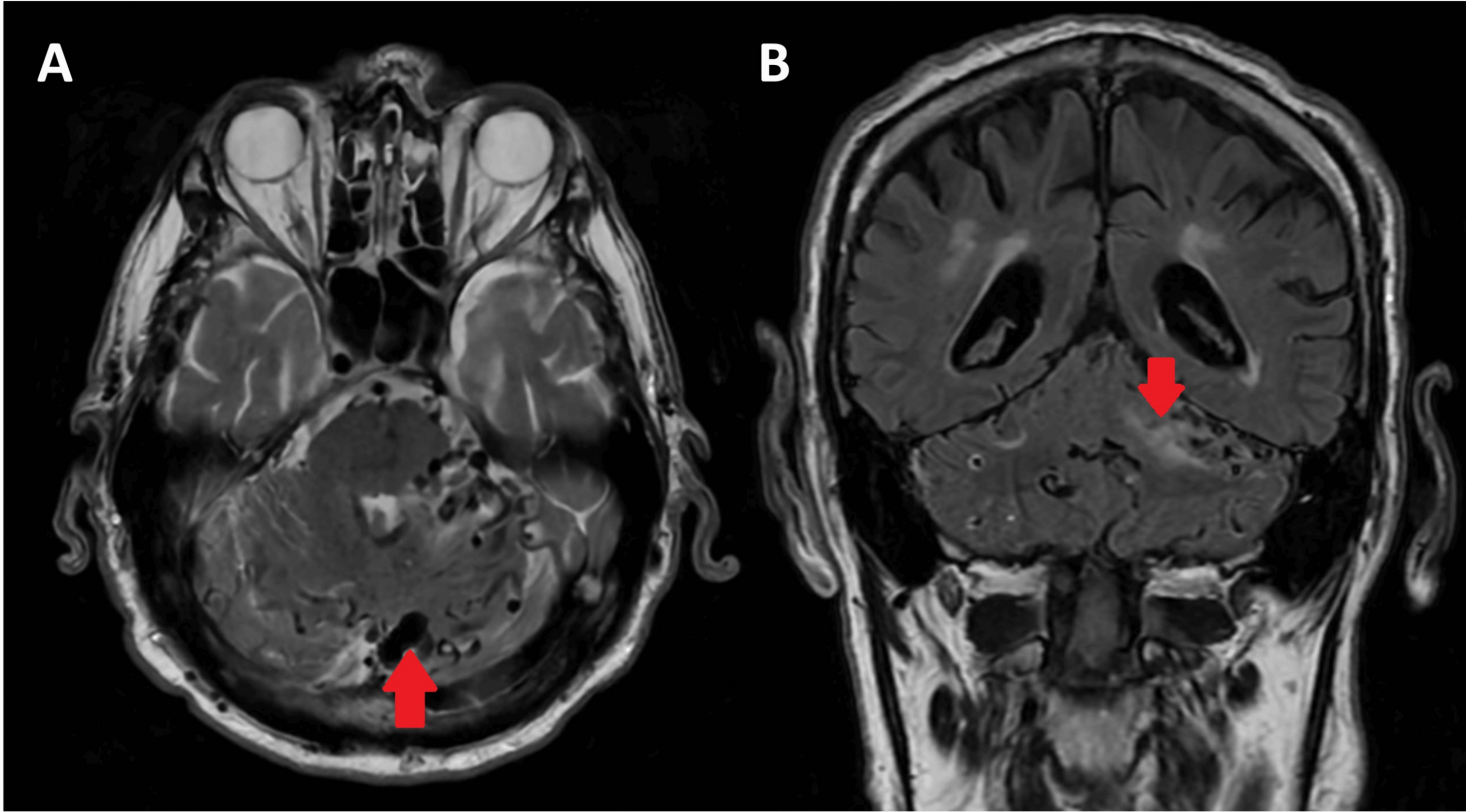
Initial MRI of the patient’s head. (A) Axial T2-weighted imaging demonstrates numerous dilated veins in the cerebellar hemispheres, vermis (red arrow), and pons. (B) Coronal fluid-attenuated inversion recovery (FLAIR) imaging demonstrates high-signal oedema (red arrow) in the left cerebellar hemisphere.

**Figure 2:**
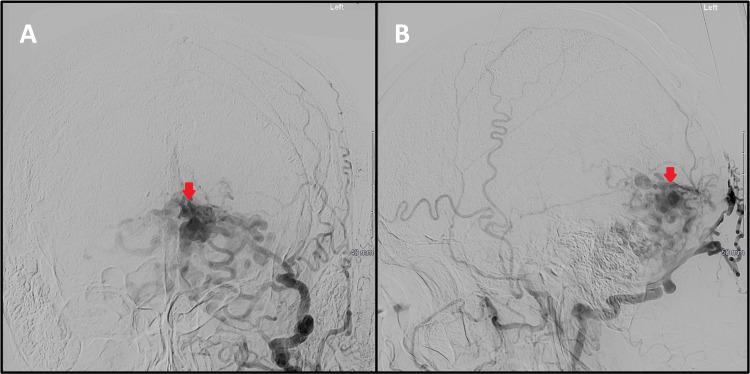
Digital subtraction angiography with injection of contrast into the left external carotid artery. The anterior-posterior (A) and lateral (B) projections demonstrate a cerebellar dural arteriovenous fistula with arterial supply from the left middle meningeal and occipital arteries. Direct cortical venous drainage into a dilated vermian vein and cerebellar hemisphere veins is noted, consistent with Cognard grade IV. The site of the arteriovenous shunt is indicated by the red arrows.

**Figure 3:**
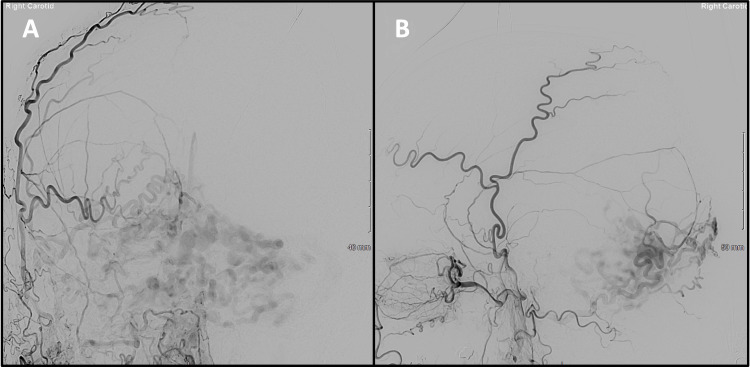
Digital subtraction angiography with injection of contrast into the right external carotid artery. The anterior-posterior (A) and lateral (B) projections demonstrate similar arterial supply from the right middle meningeal and occipital arteries.

A male in his 70s presented with several months of unsteadiness, which culminated in a fall that left him bed-bound. There were no other systemic symptoms. Neurological examination demonstrated reduced power in the lower limbs (worse on the right side), dysdiadochokinesia in the upper limbs (worse on the right side), and bilateral tremor of the upper limbs when outstretched. No other cerebellar signs were apparent. Initial CT revealed unusual dispersed calcifications within the cerebellar hemispheres but no acute intracranial findings, including focal haemorrhage. Differential diagnoses included DAVF, cavernoma, and arteriovenous malformation. MRI demonstrated dilated cerebellar and perimesencephalic veins (Figure 1). Subsequent digital subtraction angiography confirmed a high-grade (Cognard grade IV) tentorial DAVF, located just to the left of the midline at the superior vermis. The fistula had extensive arterial supply from both external carotid arteries, the left internal carotid artery, and the right vertebral artery, with venous drainage into ectatic cerebellar vermian and hemispheric veins (Figures 2-3). Endovascular embolisation was performed via the distal temporal branch of the left middle meningeal artery using a Marathon microcatheter. The plug-and-push technique was employed, which involved forming a proximal plug following initial reflux with Squid-18, followed by subsequent injection of Squid-12, allowing deeper penetration across the arteriovenous junction and into the numerous arterial feeders. During the 90-minute embolisation, Squid did not reflux beyond the initial 2-3 cm plug.

**Figure 4:**
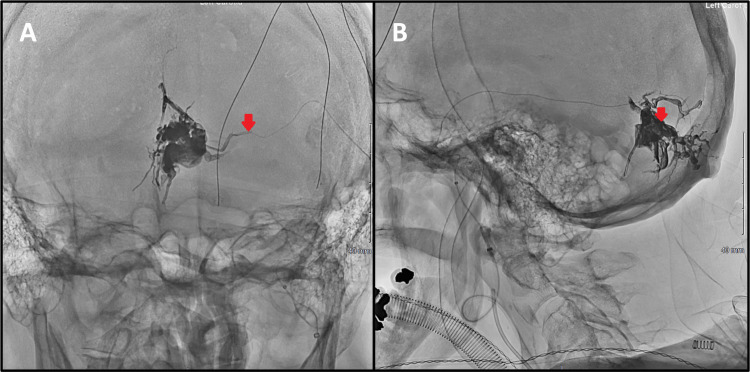
Post-embolisation digital subtraction angiography native images (no injection of contrast). The anterior-posterior projection (A) demonstrates the Squid-18 plug in the parent vessel (a branch of the left middle meningeal artery), with the extent of reflux indicated by the red arrow. Once the initial plug was formed, no further reflux was seen throughout the 90-minute embolisation. The tip of the microcatheter on the lateral projection (B) is indicated by the red arrow.

**Figure 5:**
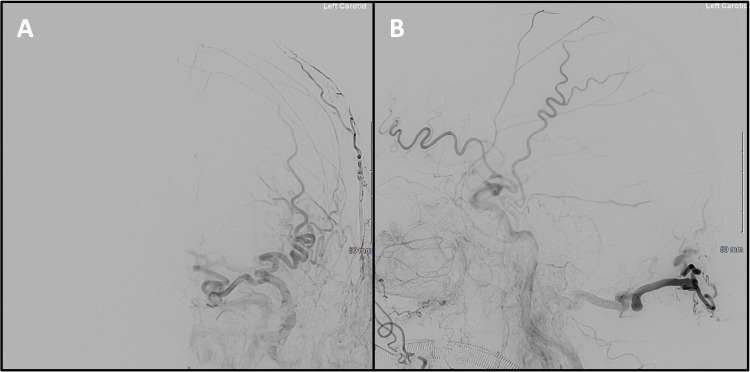
Post-embolisation control digital subtraction angiography with injection of contrast into the left external carotid artery. The anterior-posterior (A) and lateral (B) projections illustrate complete obliteration of the dural arteriovenous fistula.

Selective bilateral angiography confirmed complete occlusion of the fistula (Figures 4-5). Routine on-table CT of the head showed no post-embolisation complications. Following embolisation, the patient’s mobility improved significantly, and he was able to mobilise successfully with a frame. He was discharged home two weeks post-embolisation with a temporary reablement package and continued community physiotherapy. Eight months later, follow-up angiography demonstrated complete resolution of the dilated venous channels and DAVF, and the patient reported being well, with only occasional episodes of dizziness, but no further falls or acute concerns.

## Discussion

The plug-and-push technique has become a widely adopted strategy for EVOH-based liquid embolisation of fistulous networks, with interventionalists commonly establishing an initial anti-reflux plug using a higher-viscosity agent such as Onyx-34 and subsequently transitioning to a lower-viscosity agent (Onyx-18) to facilitate controlled distal penetration [5]. Although Onyx and Squid are both EVOH-based liquid embolic agents, Squid-12 offers an even lower-viscosity formulation that may be advantageous for enhanced antegrade flow and deeper penetration of complex vascular networks [3,6]. However, the literature to date regarding Squid remains limited and is largely confined to retrospective series and technical reports, with few studies specifically dedicated to evaluating the use of staged, viscosity-based embolisation through the plug-and-push technique described with Onyx [3].

Although this case report demonstrates the technical feasibility of this approach, its single-patient design limits conclusions regarding wider reproducibility, and additional cases are required to establish validity. Furthermore, there is currently insufficient evidence to conclude that Squid is more cost-effective than other liquid embolic agents such as Onyx or PHIL, and liquid embolic agents generally remain more expensive than traditional particulate embolic agents [7]. Alternative methods of embolisation, including dual-lumen balloon microcatheterisation, also warrant discussion. In this technique, balloon inflation is used to prevent reflux instead of a high-viscosity liquid, and therefore no plug formation is required. This approach is advantageous because it is associated with shorter procedure time, although catheter equipment is more costly and limited in availability [8].

## Conclusions

This case demonstrates the efficacy of using Squid liquid embolic agents of different viscosities with the plug-and-push technique to achieve complete occlusion of a hypervascular tentorial DAVF. The combined use of Squid-18 for proximal plug formation and Squid-12 facilitated complete penetration of the extensive fistulous network without reflux. Follow-up at eight months after the intervention confirmed the long-term durability of the occlusion on imaging, along with significant improvement in clinical symptoms. While staged viscosity-based embolisation has been described using other EVOH-based agents, this report highlights the potential applicability of a similar strategy using Squid formulations. Larger case series and comparative studies will be required to determine whether this approach offers technical or clinical advantages over established liquid embolic agents.
